# Modulating NHC catalysis with fluorine

**DOI:** 10.3762/bjoc.9.316

**Published:** 2013-12-06

**Authors:** Yannick P Rey, Ryan Gilmour

**Affiliations:** 1 Organisch-Chemisches Institut, Westfälische Wilhelms-Universität Münster, Corrensstraße 40, 48149 Münster, Germany; 2 Department of Chemistry and Applied Biosciences, ETH Zürich, Wolfgang-Pauli-Str. 10, 8093 Zürich, Switzerland,; 3 Excellence Cluster EXC 1003, Cells in Motion, Münster, Germany

**Keywords:** catalysis, enantioselectivity, fluorine, gauche effect, organo-fluorine, Steglich rearrangement

## Abstract

Fluorination often confers a range of advantages in modulating the conformation and reactivity of small molecule organocatalysts. By strategically introducing fluorine substituents, as part of a β-fluoroamine motif, in a triazolium pre-catalyst, it was possible to modulate the behaviour of the corresponding *N*-heterocyclic carbene (NHC) with minimal steric alterations to the catalyst core. In this study, the effect of hydrogen to fluorine substitution was evaluated as part of a molecular editing study. X-ray crystallographic analyses of a number of derivatives are presented and the conformations are discussed. Upon deprotonation, the fluorinated triazolium salts generate catalytically active *N*-heterocyclic carbenes, which can then participate in the enantioselective Steglich rearrangement of oxazolyl carbonates to *C*-carboxyazlactones (e.r. up to 87.0:13.0).

## Introduction

**Figure 1:**
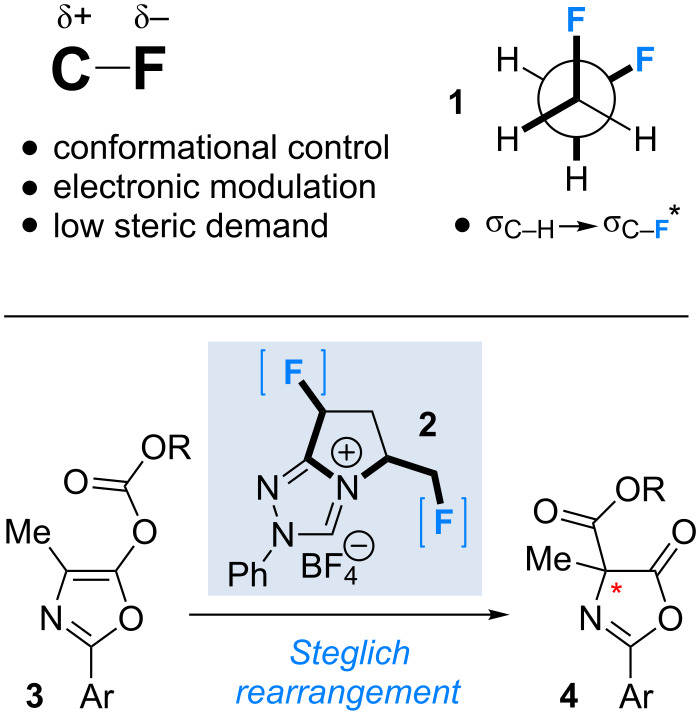
Exploring the effect of fluorine incorporation in triazolium pre-catalysts (**2**) for the enantioselective Steglich rearrangement of oxazolyl carbonates to the respective *C*-carboxyazlactones (**3**→**4**).

Molecular editing using fluorine is a powerful strategy to modulate the conformation and reactivity of small molecule organocatalysts [1–3]. The negligible steric penalty associated with H→F substitution, together with the polarised nature and stability of aliphatic C–F bonds, render this unit attractive from the perspective of molecular design [4]. The low-lying antibonding orbital (σ_C–F_*) can interact with an array of vicinal substituents ranging from non-bonding electron pairs, such as in the case of the fluorine anomeric effect [5], to electron rich sigma bonds (σ→σ*). The stereoelectronic gauche effect in 1,2-difluoroethane is the most prominent example (**1**; Figure 1) [6–9]. The counterintuitive preference of vicinal fluorine substituents to adopt a gauche preference (Φ_F–C–C–F_ ≈ 60°) can be rationalised by invoking two stabilising hyperconjugative interactions (σ_C–H_→σ_C–F_*). This conformational preference is conserved in numerous systems in which one of the fluorine atoms has been substituted by another electron withdrawing group (X^(δ+)^; X^(δ+)^–C_α_–C_β_–F^δ−^). Often this modification leads to the introduction of a stabilising electrostatic component, thus enhancing the interaction: this is exemplified by the pioneering work of O’Hagan and co-workers [10–12].

In recent years, this laboratory has strategically employed the aforementioned effects in the design of functional small molecules [13–22], often for application in organocatalysis [1]. Common to these studies has been the strategic incorporation of a fluoro substituent vicinal to a catalytically active amino group. Subsequent generation of a (partial) positive charge at nitrogen generates the requisite X–C_α_–C_β_–F^δ−^ system (X = N^+^), thus providing a facile approach to controlling rotation around this bond (Φ_XCCF_ ≈ 60°). In this study, the influence of fluorination on catalyst behaviour is extended to the study of triazolium salts such as **2**, which can be converted to the respective *N-*heterocyclic carbenes (NHCs) by simple deprotonation.

Given the importance of NHCs in modern organic synthesis [23–28] it was envisaged that these systems would be intriguing candidates for investigation. Moreover, structural information gleaned from the triazolium salt pre-catalysts regarding conformation [18,22], assist in rationalising the behaviour of the NHCs generated in situ.

Herein, the synthesis and catalytic efficiency of a series of fluorinated, bicyclic triazolium salts **2** is disclosed. The effect of molecular editing by hydrogen to fluorine substitution is evaluated in the NHC-catalysed, enantioselective Steglich rearrangement of oxazolyl carbonates **3** to *C*-carboxyazlactones **4** [29], recently reported by Smith and co-workers [30–36].

**Figure 2:**
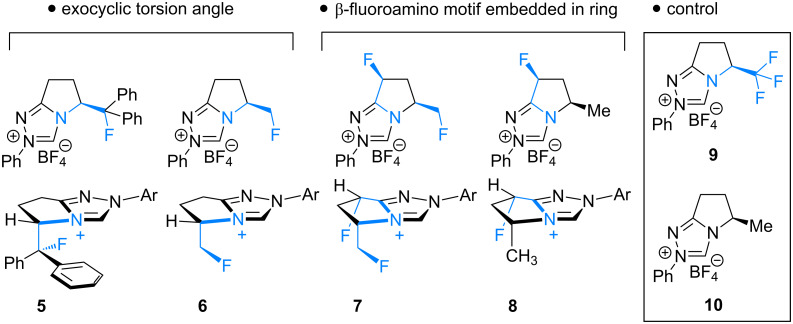
Target triazolium salts **5**–**10** for this study. The *synclinal-endo* conformation of **5** is shown [18]. Only the *synclinal-exo* arrangement of **6** and **7** is shown [22].

Fluorination sites were selected based on their proximity to the ring junction nitrogen of the triazolium system (Figure 2). Consequently, two distinct β-fluoroamine sub-classes may be generated. The first site positions the β-fluorine atom on a freely rotatable (sp^3^–sp^3^) exo cyclic group (**5**, **6** and **7**), conceivably allowing for both *synclinal-exo* and *synclinal-endo* conformations to be populated: this is consistent with the recently reported fluorine–NHC gauche effect [22]. The second fluorination site embeds the β-fluoroamine within the bicycle framework of the triazolium salt, thus restricting conformational freedom (e.g. **8**). This later scenario was inspired by the elegant work of Rovis and co-workers, which demonstrated that backbone fluorination of bicyclic NHCs improves enantioselectivity in Stetter reactions of heterocyclic aldehydes with nitroalkenes [37–40]. Finally, one hybrid system was prepared containing both β-fluoroamine classes (**7**). The trifluoromethylated triazolium salt **9** and the non-fluorinated equivalent **10** served as electronic and steric control catalysts for this study.

## Results and Discussion

### Pre-catalyst synthesis

**Scheme 1:**
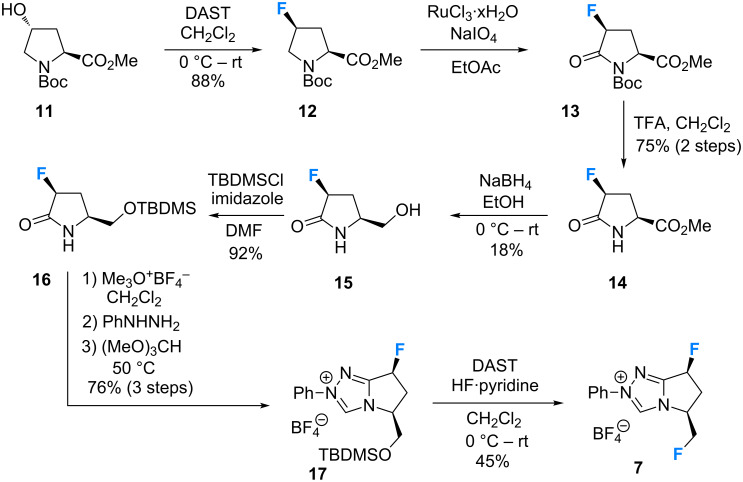
Synthesis of the difluorinated triazolium salt **7** starting from commercially available *N*-Boc-*trans*-4-hydroxy-L-proline methyl ester (**11**).

The synthesis of a novel series of fluorinated triazolium salts (**7**–**10**) is described, following our previous studies concerning the preparation of triazolium salts **5** and **6** [18,22]. The route to target **7** began by treating *N*-Boc-*trans*-4-hydroxy-L-proline methyl ester (**11**) with diethylaminosulfur trifluoride (DAST) in CH_2_Cl_2_ to install the first fluoro substituent (**12**) with clean configurational inversion (88%, Scheme 1).

Oxidation of the pyrrolidine to the corresponding lactam **13** using a Ru(III)/NaIO_4_ system proceeded smoothly, followed by TFA-mediated Boc deprotection to yield **14** (75%, 2 steps). Reduction of the methyl ester to the primary alcohol (**15**, 18%), and subsequent protection as the TBDMS ether delivered the cyclisation substrate **16** in good yield (92%). A three step, one pot sequence consisting of methylation, treatment with phenylhydrazine and subsequent cyclisation furnished the triazolium salt **17** in 76% yield (3 steps). Finally, DAST-mediated TBDMS deprotection/deoxyfluorination completed the synthetic sequence to give **7** in 45% yield.

**Scheme 2:**
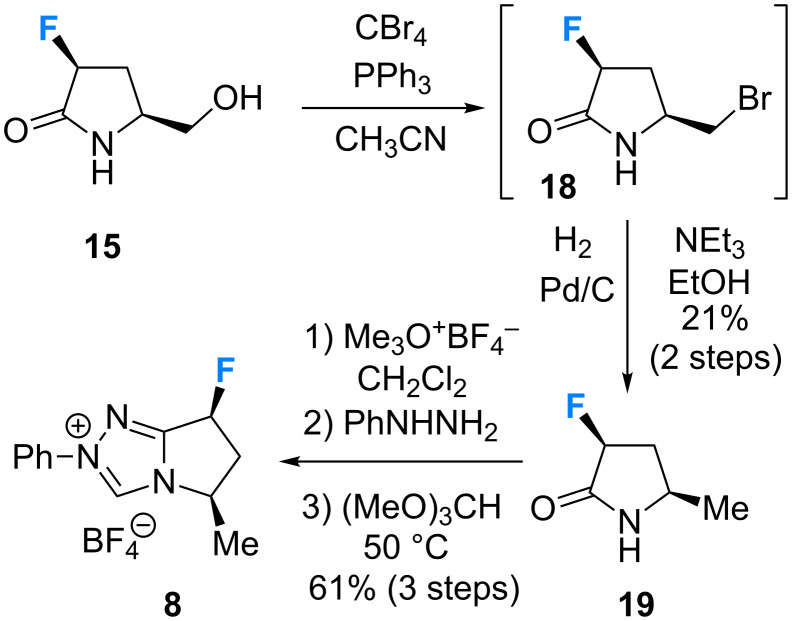
Synthesis of the monofluorinated triazolium salt **8**.

Synthesis of the monofluorinated pre-catalyst **8** (Scheme 2) commenced with an Appel reaction of alcohol **15** to prepare the primary bromide **18**. Owing to the potentially labile nature of the primary bromide, this material was used without further purification in the next step. Reduction (H_2_, Pd/C) furnished the lactam **19** (21% over 2 steps) in preparation for the cyclisation sequence. As previously described, successive treatment with the Meerwein salt, phenylhydrazine and methyl orthoformate yielded the target triazolium salt **8** in 61% over 3 steps.

**Scheme 3:**
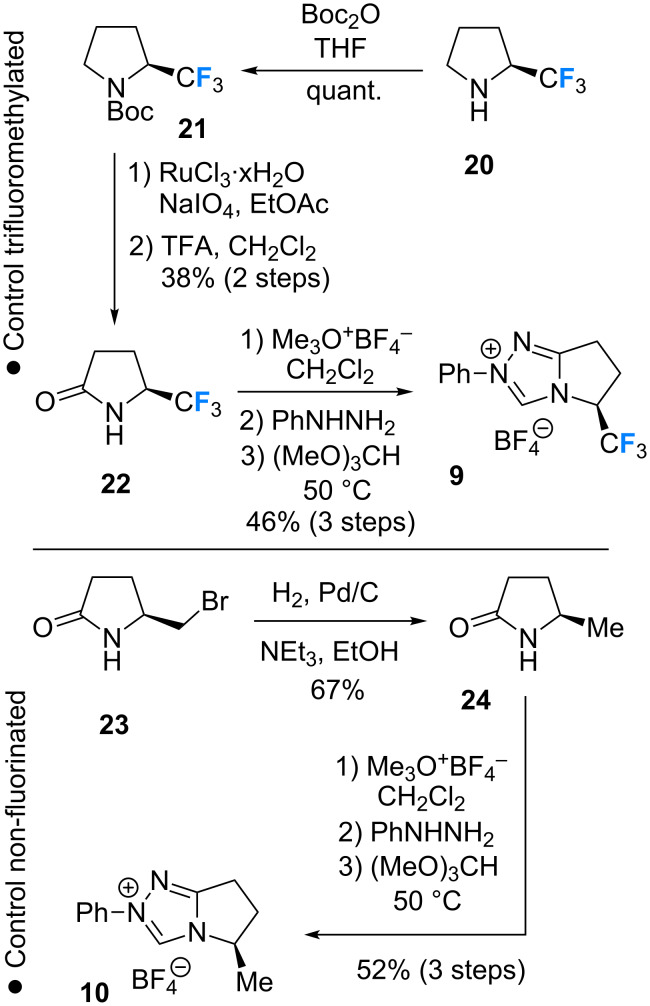
Synthesis of the trifluoromethylated and non-fluorinated pre-catalysts **9** and **10** for control studies.

The pre-catalysts **9** and **10** required for control experiments were prepared by an analogous reaction sequence (Scheme 3). Commercially available (*S*)-(+)-(trifluoromethyl)pyrrolidine **20** was protected (**21**, quantitative), oxidised to the corresponding lactam (**22**, 38% over 2 steps) and processed to the target triazolium salt **9** (46%, 3 steps). The non-fluorinated catalyst **10** (Scheme 3; lower) was prepared in a short synthesis starting from the primary bromide **23** [22]. Hydrogenolysis (**24**, 67%) [41] and subsequent conversion to the triazolium salt completed the short synthesis (52% over 3 steps).

### X-Ray structural analysis of **5**, **6** and **7**

**Figure 3:**
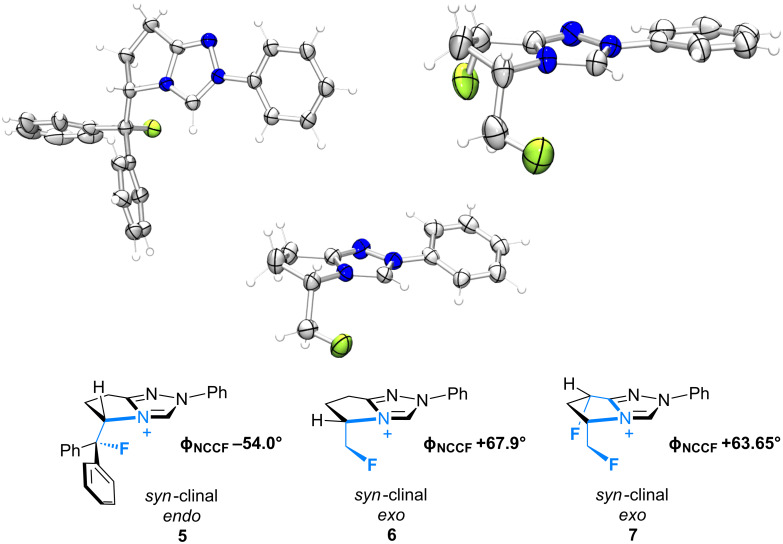
X-ray crystal structures of triazolium salts **5**·BF_4_^−^, **6**·BF_4_^−^ and **7**·BF_4_^−^ [42]. The tetrafluoroborate counterions have been omitted for clarity.

The X-ray crystal structures of triazolium salts **5**, **6** and **7** were then compared to examine the conformation of the β-fluoroamine motifs that were the major motivation for this study (Figure 3) [42]. In previous analyses of (*S*)-2-(fluorodiphenylmethyl)pyrrolidine derivatives, the *synclinal-endo* conformation was almost exclusively observed in the solid state [13,15–16,18,21–22]. This was also found to be the case in triazolium salt **5** (Φ_NCCF_ −54.0°), with the diphenylfluoromethyl group adopting a *quasi*-equatorial orientation, presumably to minimise non-bonding interactions as a consequence of the sterically demanding phenyl groups.

Deletion of these Ph units from the exocyclic group (**6**) resulted in a switch to the *synclinal-exo* conformation (Φ_NCCF_ +67.9°), with the monofluoromethyl group occupying a *quasi*-axial orientation. Interestingly, this *synclinal-endo* → *synclinal-exo* switch is also observed in the corresponding pyrrolidino systems [13,21]. The hybrid structure **7** containing both β-fluoroamine types again showed the *synclinal-exo* arrangement (Φ_NCCF_ +63.65°) as expected, although the fluorine group on the ring system did little to alter the conformation when compared with **5** and **6**.

Having completed the synthesis of the fluorinated triazolium salts (**5**–**10**) for this study, their effectiveness in catalysing the Steglich rearrangement of an oxazolyl carbonate derivative (**25**) to the corresponding *C*-carboxyazlactone **26** was investigated (Table 1). For this initial study, the monofluorinated triazolium salt **6** was arbitrarily chosen (10 mol %), with toluene being used as the reaction medium and KHMDS as the base [30]. Gratifyingly, complete conversion was observed after 18 h and with good levels of enantioselectivity (e.r. 80.5:19.5). Variation in the choice of solvent proved detrimental to both the conversion and enantioselectivity (Table 1, entries 2–8). Chlorinated solvents such as CH_2_Cl_2_ and CDCl_3_ (Table 1, entries 2 and 3) led to losses in enantioselectivity, whilst THF completely suppressed the reaction (Table 1, entry 4, <1% conversion). Intriguingly, switching from THF to Et_2_O (Table 1, entry 5) resulted in full conversion and gave appreciable enantioselectivity (e.r. 74.0:26.0). This behaviour was also preserved with 1,4-dioxane as solvent (e.r. 74.5:25.5, Table 1, entry 6). Hexane gave comparable levels of enantioinduction but with a marked decrease in conversion (38%, Table 1, entry 7). Employing chlorobenzene did not improve conversion, or the enantiomeric ratio of the products (Table 1, entry 8). Having identified toluene as the solvent of choice, attention was turned to exploring the effect of the base. It was noted that neither DBU (Table 1, entry 9) nor KO*t*-Bu (Table 1, entry 10) led to higher levels of enantioselectivity. A control reaction using solid KHMDS, rather than the commercial 0.5 M solution in toluene, revealed a lower conversion but did not alter the enantiomeric ratio (Table 1, entry 11). However, a commensurate performance was noted with Cs_2_CO_3_ (Table 1, entry 12, >99%, e.r. 80.5:19.5). Alterations in reaction concentration had little influence on the selectivity (Table 1, entries 13 and 14, 0.02 or 0.5 mol·L^−1^, e.r. 80.5:19.5 and 79.0:21.0, respectively). However, catalyst loading did dramatically alter the selectivity outcome (Table 1, entries 15–17). Given that similar enantioselectivities were recorded in reactions using Cs_2_CO_3_ (cf. KHMDS), an analogous set of reactions were run for completeness (Table 1, entries 18–21). Again, reactions using Cs_2_CO_3_ were not sensitive to changes in concentration (Table 1, entries 18 and 19), but in contrast to reactions employing KHMDS, altering the catalyst loading did not result in an erosion of the enantioselectivity (Table 1, entries 20 and 21, e.r. 80.0:20.0).

**Table 1:** Optimisation studies using triazolium salt **6**.^a,b^

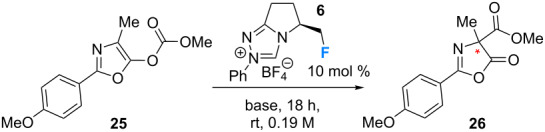							

Entry	Solvent	Base	Conc. (mol·L^−1^)	Loading (mol %)	T (°C)	Conversion (%)^b^	e.r.^b^

1	toluene	KHMDS	0.19	10	rt	>99	80.5:19.5
2	CH_2_Cl_2_	KHMDS	0.19	10	rt	89	59.0:41.0
3	CDCl_3_	KHMDS	0.19	10	rt	39	70.0:30.0
4	THF	KHMDS	0.19	10	rt	<1	—
5	Et_2_O	KHMDS	0.19	10	rt	>99	74.0:26.0
6	1,4-dioxane	KHMDS	0.19	10	rt	95	74.5:25.5
7	*n*-hexane	KHMDS	0.19	10	rt	38	79.0:21.0
8	PhCl	KHMDS	0.19	10	rt	65	71.5:28.5
9	toluene	DBU	0.19	10	rt	67	79.5:20.5
10	toluene	KO*t*-Bu	0.19	10	rt	14	78.5:21.5
11	toluene	KHMDS (solid)	0.19	10	rt	66	79.0:21.0
12	toluene	Cs_2_CO_3_	0.19	10	rt	>99	80.5:19.5
13	toluene	KHMDS	0.02	10	rt	97	80.5:19.5
14	toluene	KHMDS	0.50	10	rt	>99	79.0:21.0
15	toluene	KHMDS	0.19	30	rt	>99	66.5:33.5
16	toluene	KHMDS	0.19	5	rt	63	80.5:19.5
17	toluene	KHMDS	0.19	1	rt	<1	—
18	toluene	Cs_2_CO_3_	0.02	10	rt	92	81.0:19.0
19	toluene	Cs_2_CO_3_	0.50	10	rt	99	80.0:20.0
20	toluene	Cs_2_CO_3_	0.19	30	rt	99	80.0:20.0
21	toluene	Cs_2_CO_3_	0.19	5	rt	96	80.0:20.0

^a^Representative reaction protocol: To a suspension of **6** in the appropriate solvent was added the base indicated. The mixture was then stirred for 15 min before a solution of **25** (20.0 mg, 76.0 µmol) in toluene was added. The mixture was stirred for a further 18 h, after which time the solution was concentrated in vacuo and filtered over a plug of silica gel (CH_2_Cl_2_ as eluent). The resulting solution was then concentrated in vacuo. ^b^The conversion and enantiomeric ratio of the product were determined by HPLC on an Agilent 1260 series system using a reprocil chiral-OM 4.6 mm column. Percent conversion was determined by integration of the starting material and product peaks, correcting for response factors.

Having evaluated a series of parameters for the catalytic Steglich rearrangement using catalyst **6**, efforts were then focussed on a logical process of molecular editing to clarify the effect of H→F substitution (Table 2). Once again, toluene was employed as solvent, and reactions were run at rt for 18 h at a concentration of 0.19 M. Due to the similar enantioselectivities observed when using KHMDS and Cs_2_CO_3_ (Table 1), it was deemed prudent to perform the study using both bases independently. Initially, the bulky diphenylfluoromethyl-containing triazolium salt **5** was subjected to the optimised conditions. It was envisaged that one of the phenyl rings might assist in the facial discrimination of the activated electrophile, as a consequence of the fluorine gauche effect (Φ_NCCF_ −54.0°, Figure 3). However, the product *C*-carboxyazlactone **26** was isolated essentially in racemic form (Table 2, entry 1, e.r. 54.5:45.5). Despite the modest selectivity, the sense of induction was inverted relative to what had been previously observed. Puzzlingly, reactions in the presence of KHMDS did not yield any of the desired product (Table 2, entry 2). As previously established, deletion of the phenyl rings resulted in a marked improvement with both the conversion and enantioselectivity reaching useful values (99%, e.r. 80.5:19.5, Table 2, entries 3 and 4). As had been reported by Rovis et al. for certain Stetter reactions [37–40], fluorination of the bicycle framework (**7**) augmented the catalyst performance (Table 2, entries 5 and 6). In the presence of Cs_2_CO_3_ almost quantitative conversion was noted together with the highest enantioselectivities of the study (up to e.r. 87.0:13.0). Again, a significant loss in conversion was observed in reactions performed with KHMDS (Table 2, entry 6, 14%). Deletion of the fluorine substituent on the exo cyclic group (**8**) resulted in a notable drop in the enantiomeric ratio (77.5:22.5, Table 2, entry 7), with reactions containing KHMDS reaching only 54% conversion. Finally, in the control reaction with the trifluoromethyl-containing triazolium salt **9**, no conversion was observed irrespective of the base employed (Table 2, entries 9 and 10). Deletion of both fluorine atoms from the catalyst core (**10**) was accompanied by a drop in enantioselectivity (e.r. 62.5:37.5), although the reactions did not display the same sensitivity to changes in base (Table 2, entries 11 and 12).

**Table 2:** A catalyst molecular editing study.^a,b^

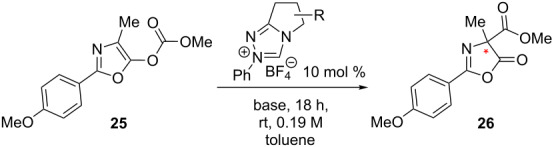				

Entry	Catalyst	Base	Conversion (%)^b^	e.r.^b^

1 2	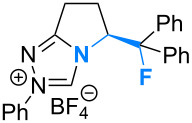 **5**	Cs_2_CO_3_ KHMDS	83 0	54.5:45.5^c^ n.d.
3 4	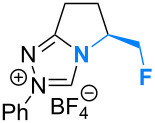 **6**	Cs_2_CO_3_ KHMDS	99 99	80.5:19.5 80.5:19.5
5 6	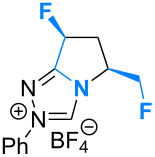 **7**	Cs_2_CO_3_ KHMDS	99 14	87.0:13.0 86.0:14.0
7 8	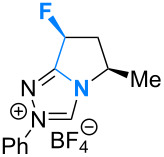 **8**	Cs_2_CO_3_ KHMDS	97 54	77.5:22.5 76.0:24.0
9 10	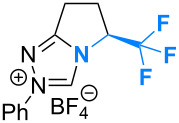 **9**	Cs_2_CO_3_ KHMDS	0 0	n.d. n.d.
11 12	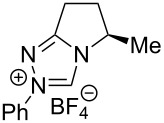 **10**	Cs_2_CO_3_ KHMDS	98 81	62.5:37.5 62.5:37.5

^a^Representative reaction protocol: A suspension of the catalyst (7.6 µmol) in toluene (200 µL) was treated with Cs_2_CO_3_ (2.5 mg, 7.6 µmol) and stirred for 15 min. A solution of **25** (20.0 mg, 76.0 µmol) in toluene (200 µL) was then added. The mixture was stirred for a further 18 h, after which time the solution was concentrated in vacuo and filtered over a plug of silica gel (CH_2_Cl_2_ as eluent). The resulting solution was then concentrated in vacuo. ^b^The conversion and enantiomeric ratio of the product were determined by HPLC on an Agilent 1260 series system using a reprocil chiral-OM 4.6 mm column. Percent conversion was determined by integration of the starting material and product peaks, correcting for response factors. ^c^Reversal in the sense of enantioselectivity.

## Conclusion

**Figure 4:**
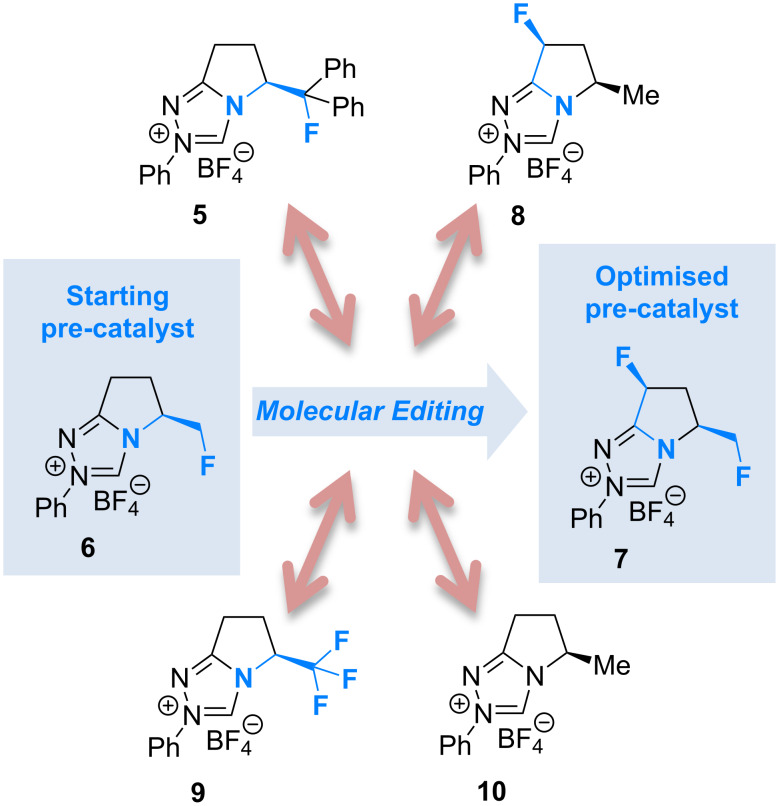
An overview of the molecular editing approach to catalyst development.

In conclusion, the ability of fluorine to modulate the catalytic performance of *N*-heterocyclic carbenes in the Steglich rearrangement of oxazolyl carbonates has been demonstrated. A focussed molecular editing study (Figure 4) has revealed that the introduction of a single fluorine atom on the exocyclic unit leads to enhanced enantioselectivities (**6** versus **10**, e.r. 80.5:19.5 versus 62.5:37.5). Further augmentation can be achieved by introduction of a second fluorine substituent on the catalyst core (**7**; e.r. 87.0:13.0, 99% conversion). However, the reinforcing role of these two fluorine substituents in orchestrating enantioinduction requires clarification and will be the subject of future investigations. What is apparent is that fluorine incorporation can confer significant advantages in (organo)catalyst optimisation and design.

## Experimental

Full experimental data is provided in Supporting Information File 1.

## Supporting Information

File 1 Experimental part.
