# Supplementary material for: Modulating NHC catalysis with fluorine
Source: Beilstein J Org Chem. 2013 Dec 6;9:2812–20. doi: 10.3762/bjoc.9.316 (PMC3869272; doi:10.3762/bjoc.9.316)

Supporting Information

for

Modulating NHC catalysis with fluorine

Yannick P. Rey[1,2] and Ryan Gilmour[1,3]*

Address: [1]Organisch-Chemisches Institut, Westfälische Wilhelms-Universität Münster, Corrensstrasse 40, 48149 Münster, Germany, [2]Department of Chemistry and Applied Biosciences, ETH Zürich, Wolfgang-Pauli-Str. 10, 8093 Zürich, Switzerland, and [3]Excellence Cluster EXC 1003, *Cells in Motion*, Münster, Germany.

Email: Ryan Gilmour - ryan.gilmour@uni-muenster.de

* Corresponding author

Experimental part

Table of Contents

General methods **S2**

Synthesis of the triazolium salts **S3**

Selected HPLC chromatograms **S14**

Selected ^{1}H, ^{13}C and ^{19}F NMR spectra of compounds 7, 8, 9, 10 and 26 **S16**

General methods

All reactions were performed under an atmosphere of argon in dried glassware, except when using aqueous reagents. All chemicals were reagent grade and used as supplied unless stated otherwise. All reactions were magnetically stirred. Solvents for extractions and chromatography were technical grade. Extracts were dried over technical grade Na_2SO_4 or $MgSO_4$. Analytical thin layer chromatography (TLC) was performed on pre-coated Merck silica gel 60 F_{254} plates (0.25 mm) and visualised by UV, CAM, ninhydrine or $KMnO_4$ stain. Flash column chromatography was carried out on Fluka silica gel 60 (230–400 mesh). Concentration in vacuo was performed at ≈10 mbar and 40 °C, drying at ≈10^{-2} mbar and room temperature (caution: some intermediates and products are volatile). ^{1}H NMR, ^{13}C NMR and ^{19}F NMR spectra were recorded on a Bruker AVANCE 300 MHz, Bruker AV 400 MHz, DRX 400 MHz, and an Agilent DD2 600 spectrometer. Chemical shifts (δ) are reported in ppm relative to the solvent residual peak. The multiplicities are reported as: s = singlet, d = doublet, t = triplet, q = quartet, sext = sextett, m = multiplet, br = broad. Melting points were measured on a Büchi B540 melting point apparatus and are uncorrected. IR spectra were measured on a Perkin-Elmer Spectrum 100 FTIR spectrometer and reported in cm^{-1}. The intensities of the bands are reported as: w = weak, m = medium, s = strong. Optical rotations were obtained using a JASCO P-2000 polarimeter in a 10 cm long cell. High-resolution mass spectra (HR ESI and EI MS) were performed by the MS service at the Laboratory of Organic Chemistry of the ETH Zürich and the Organic Chemistry Institute of the WWU Münster. HPLC analyses were performed on an Agilent 1260 system.

(2*S*,4*S*)-Methyl 4-fluoro-5-oxopyrrolidine-2-carboxylate (14)

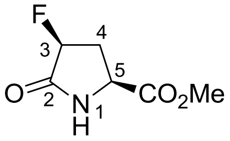

14

To a black suspension of ruthenium(III) chloride hydrate (158 mg, ≈704 µmol) in a solution of **12** (870 mg, 3.52 mmol) in EtOAc (15 mL) was added an aqueous solution of $NaIO_4$ (10%, 100 mL) to give a dark red, biphasic solution, which turned into a bright yellow solution overnight. After 3.5 days the solution was cooled to 0 °C and iPrOH (50 mL) was added to give a dark brown suspension. This was allowed to warm to rt and stirred for 7 h. Water (100 mL) was added and the mixture was extracted with EtOAc (6 × 200 mL). The combined organic layers were dried over $MgSO_4$ and concentrated in vacuo to give a white solid (664 mg). ^{1}H and ^{19}F NMR showed clean conversion with partial deprotection (protected:unprotected = 1:1.2). This mixture was dissolved in CH_2Cl_2 (35 mL) and treated with TFA (3.5 mL) drop wise. The colourless solution was stirred for 2.5 h to give a light red solution which was cooled to 0 °C. $NaHCO_3$ (sat., 50 mL) was added slowly until pH 8 was reached. The organic layer was washed and the aqueous layer was extracted with EtOAc (3 × 50 mL). The combined organic layers were dried over $MgSO_4$ and concentrated to give a light yellow oil (424 mg, 75% over two steps); R_f 0.54 (MeOH:CH_2Cl_2 1:10); $[\alpha]_D^{20}$ −40.7 (*c* 1.00 in MeOH); ν_{max} (neat)/cm^{-1} 3252w, 2960w, 2076w, 1708s, 1440m, 1332m, 1204s, 1136s, 1094m, 1056s, 1029m, 998m, 949m, 889m, 801m, 741m, 721s; ^{1}H NMR (300 MHz, $CDCl_3$): δ = 7.29 (1H, s, N*H*), 5.06 (1H, ddd, $^2J_{HF}$ 52.2, ^3J 7.8, ^3J 6.3, C^3*H*), 4.21 (1H, ddd, ^3J 8.4, ^3J 6.6, ^3J 2.4, C^5*H*), 3.80 (3H, s, CH_3), 2.89 (1H, ddt, $^3J_{HF}$ 14.8, ^2J 14.2, ^3J 7.9, C^4*H*H), 2.36 (1H, ddt, $^3J_{HF}$ 25.8, ^2J 14.1, ^3J 6.4, C^{4}H*H*); ^{13}C NMR (75 MHz, $CDCl_3$): δ = 171.9 (d, $^2J_{CF}$ 20.5, C^2), 171.0 (*C*O_2Me), 87.2 (d, $^1J_{CF}$ 185.8, C^3), 53.1 (*C*H_3), 52.0 (d, $^3J_{CF}$ 3.5, C^5), 31.9 (d, $^2J_{CF}$ 21.1, C^4); ^{19}F NMR (282 MHz, $CDCl_3$): δ = −189.7 (dddd, ^2J 52.1, ^3J 25.6, ^3J 14.3, ^4J 1.1); [*m*/*z* (ESI) found: 184.0386 $(M+Na)^+$, $C_6H_8FNO_3Na^+$ requires 184.0386].

(3*S*,5*S*)-3-Fluoro-5-(hydroxymethyl)pyrrolidin-2-one (15)

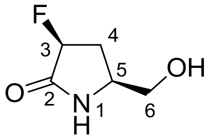

15

A light yellow solution of **14** (966 mg, 6.00 mmol) in EtOH (60 mL) was cooled to 0 °C and treated with $NaBH_4$ (454 mg, 12.0 mmol) to give a white suspension. This was stirred for 15 min and allowed to warm to rt to give a clear, colourless solution. After 90 min the solution was cooled to 0 °C and an aqueous solution of citric acid (10%, 60 mL) was added carefully. This gave a white suspension which turned back into a clear solution when the addition was complete. The solution was concentrated in vacuo to give a colourless oil, which was coevaporated with EtOH (4 × 20 mL) to give a white solid. This was purified by column chromatography (dry loading, MeOH:CH_2Cl_2 1:15) to give a white, crystalline solid (140 mg, 18%); R_f 0.21 (MeOH:CH_2Cl_2 1:10); m.p. 107–108 °C; $[\alpha]_D^{20}$ −34.8 (*c* 1.00 in MeOH); ν_{max} (neat)/cm^{-1} 3348m, 3228m, 2964w, 2945w, 2884w, 2508w, 2340w, 1980w, 1705s, 1451m, 1408m, 1330m, 1299s, 1280m, 1258w, 1233m, 1183w, 1111m, 1095s, 1075s, 1053s, 1005s, 967m, 928w, 869m, 815m, 727s, 615s; ^{1}H NMR (400 MHz, CD_3OD): δ = 5.10 (1H, ddd, $^2J_{HF}$ 53.0, ^3J 8.3, ^3J 6.7, C^3*H*), 3.75–3.55 (2H, m, C^5*H* and C^6*H*H), 3.48 (1H, dd, ^2J 11.0, ^3J 5.5, C^{6}H*H*), 2.61 (1H, dddd, $^3J_{HF}$ 13.7, ^2J 12.8, ^3J 8.3, ^3J 6.7, C^4*H*H), 1.96 (1H, ddt, $^3J_{HF}$ 28.2, ^2J 13.1, ^3J 6.5, C^{4}H*H*); ^{13}C NMR (100 MHz, CD_3OD): δ = 174.7 (d, $^2J_{CF}$ 20.6, C^2), 90.2 (d, $^1J_{CF}$ 182.6, C^3), 65.4 (C^6), 53.9 (d, $^3J_{CF}$ 3.6, C^5), 31.3 (d, $^2J_{CF}$ 19.5, C^4); ^{19}F NMR (282 MHz, CD_3OD): δ = −189.4 (dddd, ^2J 53.0, ^3J 28.8, ^3J 13.1, ^4J 4.3); [*m*/*z* (ESI) found: 156.0414 $(M+Na)^+$, $C_5H_8FNO_2Na^+$ requires 156.0431].

(3*S*,5*S*)-5-((*tert*-butyldimethylsilyloxy)methyl)3-fluoropyrrolidin-2-one (16)

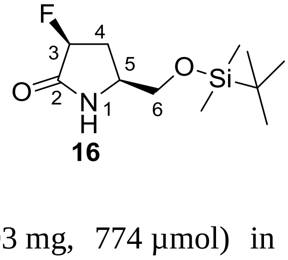

16

A colourless solution of **15** (103 mg, 774 µmol) in DMF (7.7 mL) was treated with TBDMSCl (140 mg, 929 µmol) and imidazole (79.0 mg, 1.16 mmol). The light yellow solution was stirred for 72 h and water (30 mL) was added. The mixture was extracted with

CH_2Cl_2 (3 × 30 mL). The combined organic layers were washed with water (3 × 100 mL), dried over $MgSO_4$ and concentrated. The residue was taken up in CH_2Cl_2 (30 mL) and washed with water (2 × 30 mL), dried over $MgSO_4$ and concentrated to give a white solid (176 mg, 92%); R_f 0.57 (MeOH : CH_2Cl_2 1 : 10); m.p. 78–79 °C; $[\alpha]_D^{20}$ +11.8 (*c* 0.32 in $CHCl_3$); ν_{max} (neat)/cm^{-1} 3085w, 2954m, 2930m, 2885w, 2857m, 2162w, 2051w, 1980w, 1705s, 1471w, 1462m, 1407w, 1389w, 1361w, 1337w, 1316w, 1298w, 1253m, 1183w, 1141m, 1123s, 1101s, 1084s, 1063m, 1033m, 1006m, 991m, 940w, 888w, 833s, 812s, 773s, 714s, 681m, 636m; ^{1}H NMR (400 MHz, $CDCl_3$): δ = 6.54 (1H, br s, N*H*), 5.05 (1H, ddd, $^2J_{HF}$ 52.5, ^3J 8.2, ^3J 6.5, C^3*H*), 3.73-3.63 (2H, m, C^5*H*H and C^6*H*H), 3.53–3.46 (1H, m, C^{6}H*H*), 2.57 (1H, tdd, $^3J_{HF}$ and ^2J 14.0, ^3J 8.2, ^3J 6.7, C^4*H*H), 1.91 (1H, ddt, $^3J_{HF}$ 27.1, ^2J 14.0, ^3J 6.2, C^{4}H*H*), 0.88 (9H, s, ^{t}Bu), 0.062 (3H, s, SiCH_3), 0.059 (3H, s, SiCH_3); ^{13}C NMR (100 MHz, $CDCl_3$): δ = 171.7 (d, $^2J_{CF}$ 20.7, C^2), 88.0 (d, $^1J_{CF}$ 185.0, C^3), 66.7 (C^6), 52.5 (d, $^3J_{CF}$ 3.2, C^5), 30.4 (d, $^2J_{CF}$ 19.9, C^4), 25.9 (3C, SiC(CH_3)$_3$), 18.3 (Si*C*(CH_3)$_3$), –5.32 (SiCH_3), –5.33 (SiCH_3); ^{19}F NMR (376 MHz, $CDCl_3$): δ = −187.8 (dddd, ^2J 52.4, ^3J 27.4, ^3J 13.8, ^4J 3.4); [*m/z* (ESI) found: 270.1291 $(M+Na)^+$, $C_{11}H_{22}FNO_2SiNa^+$ requires 270.1302].

(5*S*,7*S*)-5-(*tert*-Butyldimethylsilyloxy)-7-fluoro-2-phenyl-6,7-dihydro-5*H*-pyrrolo-[2,1-*c*][1,2,4]triazol-2-ium tetrafluoroborate (17)

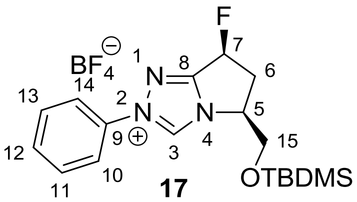

17

A colourless solution of **16** (207 mg, 838 µmol) in CH_2Cl_2 (6.0 mL) was treated with trimethyloxonium tetrafluoroborate (149 mg, 1.01 mmol) to give a suspension. After 2 h 45 min phenylhydrazine (99.3 µL, 1.01 mmol) was added to give a yellow solution. This was stirred for 15 min to give a bright orange solution which was concentrated and dried on high-vacuum (30 min) to give a sticky solid. This material was dissolved in trimethyl orthoformate (6.0 mL) and the orange solution was stirred for 29 h. The mixture was concentrated and dried on high-vacuum to give an orange solid, which was purified by column chromatography (MeOH : CH_2Cl_2 1 : 20) to give a brown solid. This was then washed with Et_2O (2 × 5 mL) to give a beige solid (277 mg, 76%); R_f 0.61 (MeOH : CH_2Cl_2 1 : 5); m.p. 119–122 °C; $[\alpha]_D^{20}$ −34.8 (*c* 0.45 in $CHCl_3$); ν_{max} (neat)/cm^{-1} 3127w, 2956w, 2931w, 2887w, 2858w, 2168w,

2052w, 1982w, 1683w, 1596w, 1525m, 1499w, 1471m, 1434w, 1412w, 1399w, 1361w, 1335w, 1320w, 1288w, 1254m, 1220m, 1208m, 1152w, 1050s, 1036s, 1005s, 976s, 940m, 915m, 836s, 776s, 759s, 723m, 712m, 685s, 667m, 611w; ^{1}H NMR (300 MHz, $CDCl_3$): δ = 10.05 (1H, s, NC*H*N), 7.83-7.73 (2H, m, C^{11}*H* and C^{13}*H*), 7.55-7.46 (3H, m, C^{10}*H*, C^{14}*H* and C^{12}*H*), 6.15 (1H, ddd, $^2J_{HF}$ 55.4, ^3J 7.6, ^3J 1.8, C^7*H*), 5.15-5.02 (1H, m, C^5*H*), 4.26 (1H, dd, ^2J 11.6, ^3J 3.2, C^{15}*H*H), 3.77 (1H, dd, ^2J 11.7, ^3J 3.6, C^{15}H*H*), 3.46 (1H, ddd, $^3J_{HF}$ 32.0, ^2J 15.6, ^3J 8.1, C^6*H*H), 2.76 (1H, ddt, $^3J_{HF}$ 27.8, ^2J 15.1, ^3J 2.4, C^6H*H*), 0.77 (9H, s, ^{t}Bu), 0.04 (3H, s, SiCH_3), 0.01 (3H, s, SiCH_3); ^{13}C NMR (75 MHz, $CDCl_3$): δ = 159.4 (d, $^2J_{CF}$ 23.5, C^8), 137.1 (N*C*HN), 135.5 (C^9), 131.2 (C^{12}), 130.4 (2C, C^{10} and C^{14}), 121.1 (2C, C^{11} and C^{13}), 83.1 (d, $^1J_{CF}$ 187.7, C^7), 62.9 (C^{15}), 61.9 (C^5), 37.5 (d, $^2J_{CF}$ 22.4, C^6), 25.7 (3C, SiC($CH_3)_3$), 18.2 (Si*C*($CH_3)_3$), −5.5 (Si*C*H_3), −5.6 (Si*C*H_3); ^{19}F NMR (282 MHz, $CDCl_3$): δ = −151.7 ($^{10}BF_4^-$), −151.8 ($^{11}BF_4^-$), −173.4 (dtd, ^2J 54.4, ^3J 27.2, ^4J 2.5); [*m*/*z* (ESI) found: 348.1913 $(M–BF_4^-)^+$, $C_{18}H_{27}FN_3OSi^+$ requires 348.1902].

(5*S*,7*S*)-5-Fluoromethyl-7-fluoro-2-phenyl-6,7-dihydro-5*H*-pyrrolo-[2,1-*c*][1,2,4]-triazol-2-ium tetrafluoroborate (7)

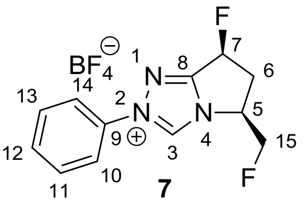

7

A light orange solution of **17** (250 mg, 575 µmol) in CH_2Cl_2 (5.8 mL) in a polypropylene flask was cooled to 0 °C and treated with hydrogen fluoride pyridine (70%, 149 µL, 5.75 mmol) dropwise to give a deep blue solution. DAST (152 µL, 1.15 mmol) was added dropwise to give a brown solution which was allowed to warm to rt and stirred for 21 h. The dark brown solution was cooled to 0 °C and carefully treated with $NaHCO_3$ (sat., 10 mL) and allowed to warm to rt. The mixture was extracted with CH_2Cl_2 (6 × 10 mL), the combined organic layers were dried over Na_2SO_4 and concentrated to give a brown solid. This was purified by column chromatography (MeOH:CH_2Cl_2 1:20) and washed with $CHCl_3$ to give a light brown solid (83.0 mg, 45%). Crystals which were suitable for X-ray analysis were obtained by vapor diffusion (Et_2O/MeOH). R_f 0.27 (MeOH:CH_2Cl_2 1:10); m.p. 143–145 °C; $[\alpha]_D^{20}$ −9.4 (*c* 0.52 in acetone); ν_{max} (neat)/cm^{-1} 3110w, 3036w, 2926w, 2855w, 2331w, 2162w, 1974w, 1706w, 1595w, 1524m, 1470w, 1433w, 1407w, 1332w, 1290w, 1258w,

1222m, 1165w, 1005s, 973s, 949s, 920m, 890m, 874m, 836w, 768s, 746m, 688s, 656m, 633w, 613w; ^{1}H NMR (400 MHz, $(CD_3)_2CO$): δ = 10.67 (1H, s, NC*H*N), 8.07–7.97 (2H, m, $C^{11}H$ and $C^{13}H$), 7.78–7.67 (3H, m, $C^{10}H$, $C^{14}H$ and $C^{12}H$), 6.51 (1H, dddd, $^{2}J_{HF}$ 54.6, ^{3}J 7.3, ^{3}J 2.3, ^{4}J 1.1, $C^{7}H$), 5.52-5.39 (1H, m, $C^{5}H$), 5.18 (1H, ddd, $^{3}J_{HF}$ 46.2, ^{2}J 10.6, ^{3}J 3.0, FC*H*H), 4.90 (1H, ddd, $^{3}J_{HF}$ 46.7, ^{2}J 10.6, ^{3}J 6.6, FCH*H*), 3.71 (1H, ddddd, $^{3}J_{HF}$ 25.7, ^{2}J 16.0, ^{3}J 8.9, ^{3}J 7.3, $^{4}J_{HF}$ 1.6, $C^{6}H$H), 2.94 (1H, dddd, $^{3}J_{HF}$ 26.9, ^{2}J 15.5, ^{3}J 3.8, ^{3}J 2.4, $C^{6}HH$); ^{13}C NMR (100 MHz, $(CD_3)_2CO$): δ = 159.9 (d, $^{2}J_{CF}$ 23.4, C^{8}), 139.9 (N*C*HN), 136.8 (C^{9}), 132.1 (C^{12}), 131.1 (2C, C^{10} and C^{14}), 122.5 (2C, C^{11} and C^{13}), 84.1 (d, $^{1}J_{CF}$ 184.4, C^{7}), 83.0 (d, $^{1}J_{CF}$ 173.3, C^{15}), 60.8 (d, $^{2}J_{CF}$ 19.2, C^{5}), 37.5 (dd, $^{2}J_{CF}$ 22.6, $^{2}J_{CF}$ 6.3, C^{6}); ^{19}F NMR (282 MHz, $(CD_3)_2CO$): δ = −151.8 ($^{10}BF_4^-$), −151.9 ($^{11}BF_4^-$), −174.9 (ddddd, ^{2}J 54.8, ^{3}J 26.8, ^{3}J 25.7, $^{5}J_{FF}$ 5.1, J 4.1, $C^{7}F$), −226.0 (tdt, ^{2}J 46.7, ^{3}J 20.3, $^{5}J_{FF}$ 4.6, $C^{15}F$); [*m/z* (ESI) found: 236.0990 $(M–BF_4^-)^+$, $C_{12}H_{12}F_2N_3^+$ requires 236.0994].

(3*S*,5*S*)-5-(Bromomethyl)-3-fluoropyrrolidin-2-one (18)

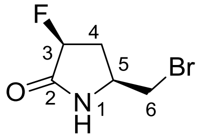

18

A white suspension of **15** (207 mg, 1.56 mmol) and triphenylphosphine (568 mg, 1.71 mmol) in acetonitrile (2.5 mL) was cooled to 0 °C and treated with a solution of tetrabromomethane (568 mg, 1.71 mmol) in acetonitrile (5.0 mL). The mixture was allowed to warm to rt to give a clear, light yellow solution. This was stirred for 5 d, concentrated in vacuo and dried on high vacuum. *n*-Hexane (5.0 mL) and water (5.0 mL) was added and the resulting suspension was vigorously stirred for 1 h and filtered. *n*-Hexane (5.0 mL) and water (5.0 mL) was added to the residue, the mixture was stirred for 30 min and filtered. The aqueous layer of the combined solutions was extracted with $CHCl_3$ (6 × 10 mL). The combined organic layers (only $CHCl_3$) were dried over Na_2SO_4 and concentrated in vacuo to give a white solid (117 mg) which was directly used in the next step. R_f 0.43 (MeOH:CH_2Cl_2 1:10); ^{1}H NMR (300 MHz, $CDCl_3$): δ = 6.51 (1H, s, N*H*), 5.06 (1H, ddd, $^{2}J_{HF}$ 52.1, ^{3}J 7.8, ^{3}J 5.7, $C^{3}H$), 3.96–3.84 (1H, m, $C^{5}H$), 3.49 (1H, dd, ^{2}J 10.4, ^{3}J 5.0, $C^{6}H$H), 3.38 (1H, dd, ^{2}J 10.4, ^{3}J 8.0, $C^{6}HH$), 2.68 (1H, ddt, $^{3}J_{HF}$ 17.4, ^{2}J 14.6, ^{3}J 7.4, $C^{4}H$H), 2.08 (1H, ddt, $^{3}J_{HF}$ 25.8, ^{2}J 14.4, ^{3}J 5.5, $C^{4}HH$); ^{19}F NMR (282 MHz, $CDCl_3$): δ = −186.2 (dddd, ^{2}J 51.9, ^{3}J 26.0, ^{3}J 17.5, ^{4}J 3.4).

(3*S*,5*R*)-3-Fluoro-5-methylpyrrolidin-2-one (19)

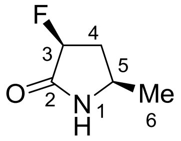

19

To a solution of **18** (102 mg, 520 μmol) and triethylamine (72 μL, 520 μmol) in EtOH (1.5 mL) was added palladium on carbon (5%, 30 mg) to give a black suspension, which was placed under an atmosphere of hydrogen (≈1 bar). This was stirred for 46 h and filtered over celite. The solution was concentrated in vacuo to give a white solid which was purified by column chromatography (MeOH:CH_2Cl_2 1:20) to give a white solid (33.6 mg, 21% over 2 steps). R_f 0.42 (MeOH:CH_2Cl_2 1:10); m.p. 104–105 °C; $[\alpha]_D^{20}$ −0.5 (*c* 1.00 in $CHCl_3$); ν_{max} (neat)/cm^{-1} 3414w, 3002w, 2976m, 2937m, 2879m, 2802w, 2755m, 2738m, 2676s, 2603m, 2529w, 2492m, 2347w, 2239w, 1981w, 1707w, 1475s, 1434s, 1397s, 1384m, 1365m, 1332w, 1288w, 1170s, 1117m, 1069m, 1035s, 904w, 849m, 804s, 750m, 719m, 690m, 622w; ^{1}H NMR (300 MHz, $CDCl_3$): δ = 7.69 (1H, br s, N*H*), 5.05 (1H, dt, $^2J_{HF}$ 52.6, ^3J 7.8, C^3H), 3.66 (1H, sextd, ^3J 6.4, $^4J_{HF}$ 3.6, C^5H), 2.68 (1H, dddd, ^2J 13.5, $^3J_{HF}$ 10.1, ^3J 8.1, ^3J 6.3, C^4HH), 1.82 (1H, ddt, $^3J_{HF}$ 26.5, ^2J 13.4, ^3J 7.4, C^4H*H*), 1.30 (3H, d, ^3J 6.2, CH_3); ^{13}C NMR (75 MHz, $CDCl_3$): δ = 172.7 (d, $^2J_{CF}$ 20.2, C^2), 89.0 (d, $^1J_{CF}$ 185.3, C^3), 46.5 (d, $^3J_{CF}$ 4.9, C^5), 36.6 (d, $^2J_{CF}$ 18.3, C^4), 22.4 (CH_3); ^{19}F NMR (282 MHz, $CDCl_3$): δ = −189.2 (dddd, ^2J 52.6, ^3J 26.5, ^3J 10.1, ^4J 3.4).

(5*R*,7*S*)-7-Fluoro-5-methyl-2-phenyl-6,7-dihydro-5*H*-pyrrolo[2,1-*c*][1,2,4]triazol-2-ium tetrafluoroborate (8)

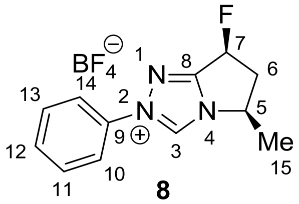

8

To a colourless solution of **19** (25.3 mg, 216 µmol) in CH_2Cl_2 (2.0 mL) was added $Me_3O^+{\cdot}BF_4^-$ (38.4 mg, 259 µmol) to give a suspension which turned into a light yellow solution over 3.5 h. Phenylhydrazine (26 µL, 259 µmol) was added to give an orange solution which was stirred for 15 min and concentrated. The resulting red solid was dissolved in trimethyl orthoformate (2.0 mL), heated to 50 °C and stirred for 14 h. The yellow solution was cooled to rt and concentrated to give an orange solid which was purified by column chromatography (MeOH:CH_2Cl_2 1:20) to give an orange solid (40.4 mg, 61%). R_f 0.16 (MeOH:CH_2Cl_2 1:10); m.p. 186–189 °C; $[\alpha]_D^{20}$ −18.2 (*c* 1.00 in acetone); ν_{max} (neat)/cm^{-1} 3133w, 2925w, 2355w, 2162w, 1980w, 1705w, 1593m, 1523m, 1497w, 1471w, 1433m, 1404m, 1395m, 1344m, 1293w, 1228s, 1184w, 1157w, 1051s, 1032s, 974s, 919s, 899m, 869m, 843w, 786w, 764s, 739s, 686s, 634m, 612m; ^{1}H NMR (300 MHz, $(CD_3)_2CO$): δ = 10.59 (1H, s, NC*H*N), 8.01–7.95 (2H, m, $C^{11}H$ and $C^{13}H$), 7.75–7.65 (3H, m, $C^{10}H$, $C^{14}H$ and $C^{12}H$), 6.47 (1H, ddd, $^2J_{HF}$ 54.6, ^3J 7.1, ^3J 3.3, C^7H), 5.23–5.09 (1H, m, C^5H), 3.64 (1H, dddd, $^3J_{HF}$ 23.1, ^2J 14.9, ^3J 7.6, ^3J 7.2, C^6HH), 2.74 (1H, dddd, $^3J_{HF}$ 26.6, ^2J 14.9, ^3J 4.5, ^3J 3.4, C^6HH), 1.84 (3H, d, ^3J 6.7, CH_3); ^{13}C NMR (75 MHz, $(CD_3)_2CO$): δ = 159.5 (d, $^2J_{CF}$ 23.4, C^8), 139.4 (NCHN), 136.9 (C^9), 131.9 (C^{12}), 131.1 (2C, C^{10} and C^{14}), 122.2 (2C, C^{11} and C^{13}), 85.0 (d, $^1J_{CF}$ 183.5, C^7), 57.6 (C^5), 43.5 (d, $^2J_{CF}$ 21.0, C^6), 21.1 (CH_3); ^{19}F NMR (282 MHz, $(CD_3)_2CO$): δ = −151.5 ($^{10}BF_4^-$), −151.6 ($^{11}BF_4^-$), −176.5 (dddd, $^2J_{HF}$ 54.2, ^3J 27.0, ^3J 23.2, $^4J_{HF}$ 4.1); [*m*/*z* (ESI) found: 218.1091 $(M\text{-}BF_4^-)^+$, $C_{12}H_{13}FN_3O^+$ requires 218.1088].

(*R*)-5-Methyl-2-phenyl-6,7-dihydro-5*H*-pyrrolo[2,1-*c*][1,2,4]triazol-2-ium tetrafluoroborate (10)

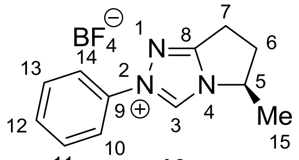

10

To a yellow solution of **24** (54.8 mg, 55.3 µmol) in CH_2Cl_2 (5.5 mL) was added $Me_3O^+{\cdot}BF_4^-$ (98.3 mg, 664 µmol) to give a suspension which turned into a colourless solution over 1 h. Phenylhydrazine was added to give a yellow solution. This was stirred for 3 h to give a bright red solution, which was concentrated in vacuo. The residue was dissolved in trimethyl orthoformate (5.5 mL) and heated to 50 °C. After 12 h the mixture was cooled to rt and concentrated in vacuo to give a brown oil. This was purified by column chromatography (MeOH:CH_2Cl_2 1:20) to give a beige solid (82.0 mg, 52%). R_f 0.21 (MeOH:CH_2Cl_2 1:10); m.p. 120–124 °C; $[\alpha]_D^{20}$ −23.1 (*c* 1.00 in acetone); ν_{max} (neat)/cm^{-1} 3133w, 2927w, 2265w, 2116w, 1981w, 1659w, 1591m, 1521m, 1497w, 1469m, 1438m, 1385m, 1318w, 1287w, 1223m, 1056s, 978m, 914s, 764s, 733s, 689m, 649m; ^{1}H NMR (400 MHz, $(CD_3)_2CO$): δ = 10.44 (1H, s, NC*H*N), 8.01-7.90 (2H, m, C^{11}*H* and C^{13}*H*), 7.74–7.59 (3H, m, C^{10}*H*, C^{14}*H* and C^{12}*H*), 5.08 (1H, sext, ^3J 6.8, C^5*H*), 3.47–3.28 (2H, m, C^7*H$_2$*), 3.14 (1H, dtd, ^2J 12.9, ^3J 7.6, ^3J 5.2, C^6*H*H), 2.60 (1H, dddd, ^2J 13.1, ^3J 9.1, ^3J 8.2, ^3J 7.5, C^{6}H*H*), 1.77 (3H, d, ^3J 6.5); ^{13}C NMR (100 MHz, $(CD_3)_2CO$): δ = 163.6 (*C*^{8}), 138.2 (N*C*HN), 137.0 (*C*^{9}), 131.4 (*C*^{12}), 131.1 (2C, *C*^{10} and *C*^{14}), 121.8 (2C, *C*^{11} and *C*^{13}), 58.5 (*C*^{5}), 36.1 (*C*^{6}), 22.5 (*C*^{7}), 19.7 (*C*H_3); ^{19}F NMR (282 MHz, $(CD_3)_2CO$): δ = −151.4 ($^{10}BF_4^-$), −151.5 ($^{11}BF_4^-$); [*m*/*z* (ESI) found: 200.1185 $(M–BF_4^-)^+$, $C_{12}H_{14}N_3^+$ requires 200.1183].

(*S*)-*tert*-Butyl 2-(trifluoromethyl)-pyrrolidine-1-carboxylate (21)

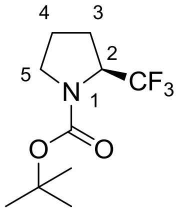

21

To a solution of **20** (178 mg, 1.28 mmol) in THF (13 mL) was added a solution of Boc_2O (279 mg, 1.28 mmol) in THF (2.0 mL) to give a colourless solution which was stirred for 15 h. CH_2Cl_2 (50 mL) was added and the mixture was washed with water (50 mL). The organic layer was dried over Na_2SO_4 and concentrated in vacuo to give a light yellow liquid (306 mg; quant.) as a mixture of rotamers. R_f 0.57 (CH_2Cl_2); $[\alpha]_D^{20}$ +7.4 (*c* 0.75 in $CHCl_3$); ν_{max} (neat)/cm^{-1} 2980w, 2894w, 2289w, 2113w, 1982w, 1810w, 1774w, 1705s, 1480w, 1457w, 1393s, 1367s, 1310m, 1288m, 1270s, 1235m, 1208m, 1163s, 1137s, 1115s, 1068s, 982m, 922m, 905m, 882m, 847m, 808m, 774m, 682m, 608w; ^{1}H NMR (600 MHz, $CDCl_3$): δ = 4.51–4.25 (1H, br m, C^2H), 3.61-3.36 (2H, br m, C^5H_2), 2.12–1.85 (4H, br m, C^3H_2 and C^4H_2), 1.46 (9H, s, *t*Bu); ^{13}C NMR (150 MHz, $CDCl_3$): δ = 154.5 (N*C*O), 126.0 (br q, $^1J_{CF}$ 282, *C*F_3), 80.7 (O*C*(CH_3)$_3$), 57.9 (C^2), 47.2 and 46.7 (C^5), 28.4 (3C, C(CH_3)$_3$), 26.6, 25.8, 23.9 and 23.0 (C^3 and C^4); ^{19}F NMR (564 MHz, $CDCl_3$): δ = −75.1 and −75.2; [*m*/*z* (ESI) found: 262.1028 (M+Na)$^+$, $C_{10}H_{16}F_3NO_2Na^+$ requires 262.1031].

(*S*)-5-(Trifluoromethyl)pyrrolidin-2-one (22)

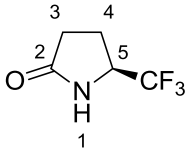

22

To a solution of **21** (247 mg, 1.03 mmol) in EtOAc (5.0 mL) was added an aqueous solution of $NaIO_4$ (10%, 30 mL) to give a biphasic mixture which was vigorously stirred. Ruthenium(III) chloride hydrate was added (47.0 mg, ≈207 µmol) to give a brown solution. After 14.5 h iPrOH (15 mL) was added to the now yellow solution to give a black suspension, which was stirred for 3 h. Water (100 mL) was added and the mixture was extracted with EtOAc (200 mL). The organic layer was dried over Na_2SO_4 and concentrated to give a brown

oil (241 mg). This was dissolved in CH_2Cl_2 (10 mL) and treated with TFA (1.0 mL). The solution was stirred for 45 min and poured into $NaHCO_3$ (sat., 50 mL). The mixture was extracted with EtOAc (50 mL), the organic layer was dried over Na_2SO_4 and concentrated in vacuo to give a yellow solid. This was purified by column chromatography (MeOH:CH_2Cl_2 1:10) to give a light yellow solid (60.0 mg, 38% over 2 steps). R_f 0.57 (MeOH:CH_2Cl_2 1:10); m.p. 104–105 °C (lit.[1] 107–108 °C); $[\alpha]_D^{20}$ +2.2 (*c* 0.66 in MeOH) (lit.[1] −5.5 (*c* 1.20 in MeOH)); ^{1}H NMR (300 MHz, $CDCl_3$): δ = 6.80 (1H, br s, N*H*), 4.14–4.00 (1H, m, NC*H*), 2.60–2.15 (4H, m, C^3H_2 and C^4H_2); ^{13}C NMR (75 MHz, $CDCl_3$): δ = 178.5 (C^2), 125.3 (q, $^1J_{CF}$ 280.5, CF_3), 55.1 (q, $^2J_{CF}$ 32.5, NCH), 28.5 (C^3), 20.8 (q, $^3J_{CF}$ 1.8, C^4); ^{19}F NMR (282 MHz, $CDCl_3$): δ = −78.8 (d, ^3J 7.0).

[1] Bezdudny, A. V.; Alekseenko, A. N.; Mykhailiuk, P. K.; Manoilenko, O. V.; Shishkin, O. V.; Pustovit, Y. M. *Eur. J. Org. Chem.* **2011**, 1782–1785.

(*S*)-2-Phenyl-5-(trifluoromethyl)-6,7-dihydro-5*H*-pyrrolo[2,1-*c*][1,2,4]triazol-2-ium tetrafluoroborate (9)

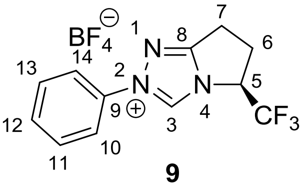

9

To a light yellow solution of **22** (50.0 mg, 327 µmol) in CH_2Cl_2 (3.0 mL) was added $Me_3O^+\cdot BF_4^-$ (58.0 mg, 392 µmol) to give a suspension. This was stirred for 75 min, treated with phenylhydrazine (39 µL, 392 µmol) and stirred for 13.5 h. The solution was concentrated in vacuo and the residue was dissolved in trimethyl orthoformate (3.0 mL). The solution was stirred at rt for 2.5 h, heated to 50 °C and stirred for 4.5 h. The yellow solution was cooled to rt and concentrated in vacuo to give a dark yellow oil. This material was purified by column chromatography (MeOH:CH_2Cl_2 1:15) to give a brown solid which was washed with $CHCl_3$ to give a beige solid (52.0 mg, 46%). R_f 0.32 (MeOH:CH_2Cl_2 1:10); m.p. 139–142 °C; $[\alpha]_D^{20}$ +117.9 (*c* 0.67 in acetone); ν_{max} (neat)/cm^{-1} 3109w, 3048w, 2981w, 2325w, 2164w, 1994w, 1760w, 1608m, 1595m, 1530m, 1514w, 1471w, 1443w, 1425w, 1383m, 1330w, 1291s, 1232w, 1179s, 1164m, 1137s, 1060s, 1048s, 1018s, 972s, 919m, 905m, 887m, 843s, 765s, 735m, 699m, 687s, 675s, 638m, 614w; ^{1}H NMR (400 MHz, $CDCl_3$): δ = 10.7 (1H, s, NC*H*N), 8.03-7.96 (2H, m, $C^{11}H$ and $C^{13}H$), 7.75–7.65 (3H, m, $C^{10}H$, $C^{14}H$ and $C^{12}H$), 5.92–5.82 (1H,

m, C^5H), 3.65–3.38 (3H, m, C^6HH and C^7H_2), 3.18–3.08 (1H, m, C^6HH); ^{13}C NMR (100 MHz, $CDCl_3$): $\delta = 164.4$ (C^8), 139.7 (NCHN), 136.8 (C^9), 132.0 (C^{12}), 131.0 (2C, C^{10} and C^{14}), 124.4 (q, $^1J_{CF}$ 279.9, CF_3), 122.5 (2C, C^{11} and C^{13}), 60.6 (q, $^2J_{CF}$ 34.7, C^5), 29.0 (q, $^3J_{CF}$ 1.5, C^6), 21.7 (C^7); ^{19}F NMR (282 MHz, $CDCl_3$): $\delta = -76.0$ (d, ^3J 7.0, CF_3), −152.0 ($^{10}BF_4^-$), −152.1 ($^{11}BF_4^-$); [m/z (ESI) found: 254.0905 $(M–BF_4^-)^+$, $C_{12}H_{11}F_3N_3O^+$ requires 254.0900].

Selected HPLC chromatograms

Table 2, entry 1

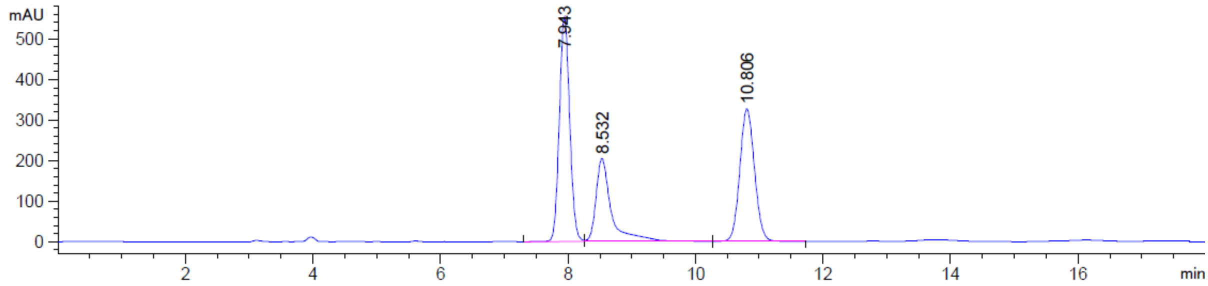

Signal 1: DAD1 B, Sig=254,4 Ref=360,100

Peak #	RetTime [min]	Type	Width [min]	Area [mAU*s]	Height [mAU]	Area %
1	7.943	BV	0.1756	6251.53027	554.35162	41.9632
2	8.532	VB	0.2448	3425.19580	205.13725	22.9915
3	10.806	BB	0.2485	5220.92041	326.66290	35.0453

Table 2, entry 5

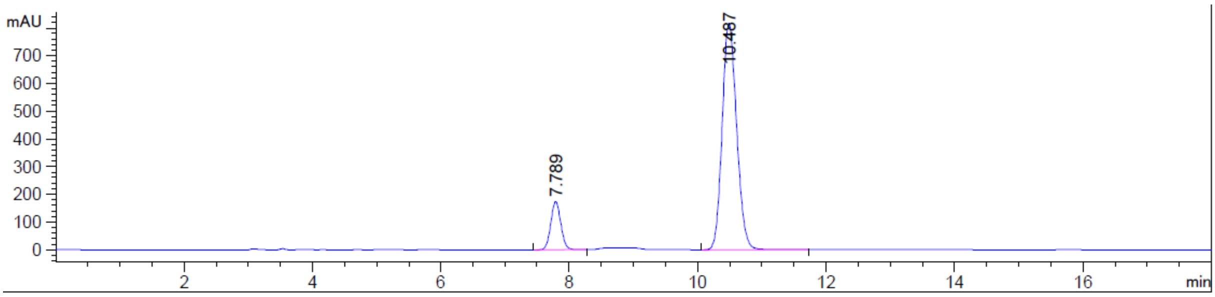

Signal 1: DAD1 B, Sig=254,4 Ref=360,100

Peak #	RetTime [min]	Type	Width [min]	Area [mAU*s]	Height [mAU]	Area %
1	7.789	BB	0.1685	1883.84290	173.73604	12.8802
2	10.487	BB	0.2421	1.27420e4	816.39978	87.1198

starting material

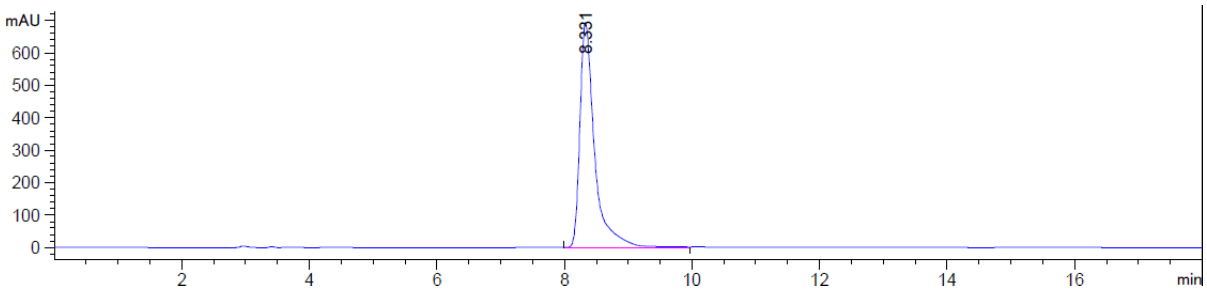

Signal 1: DAD1 B, Sig=254,4 Ref=360,100

Peak #	RetTime [min]	Type	Width [min]	Area [mAU*s]	Height [mAU]	Area %
1	8.331	BB	0.2360	1.10349e4	692.31836	100.0000

Selected NMR spectra

^{1}H NMR

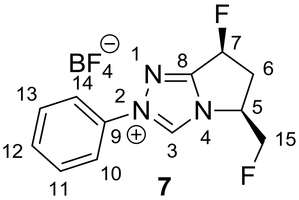

7

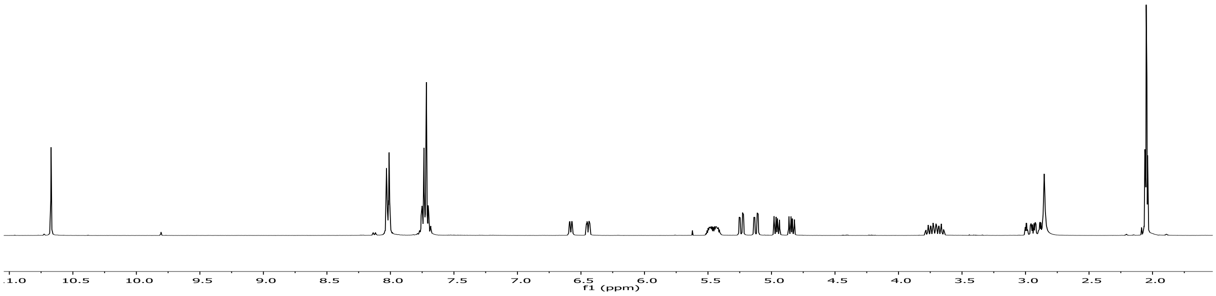

^{13}C NMR

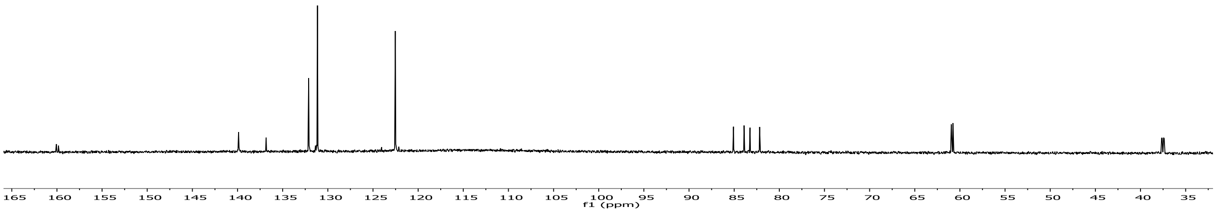

^{19}F NMR

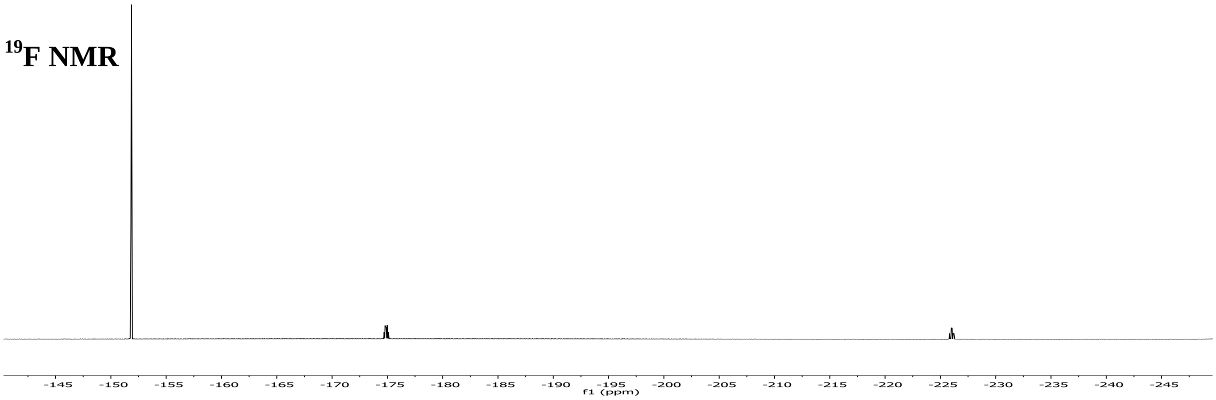

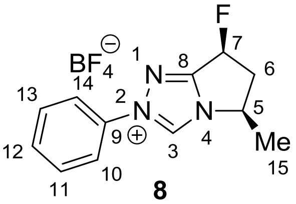

8

^{1}H NMR

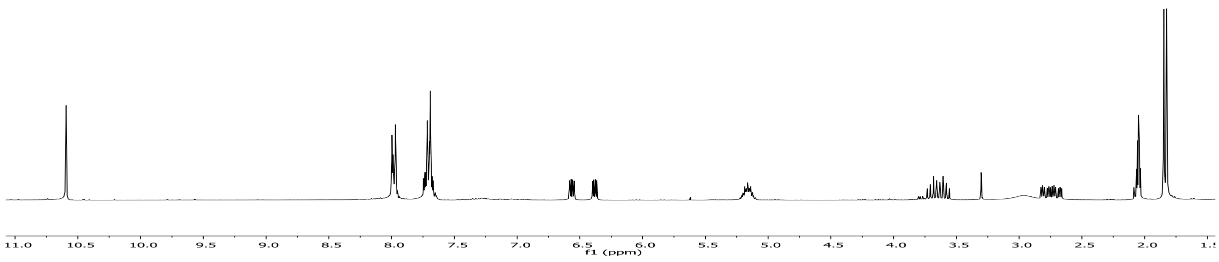

^{13}C NMR

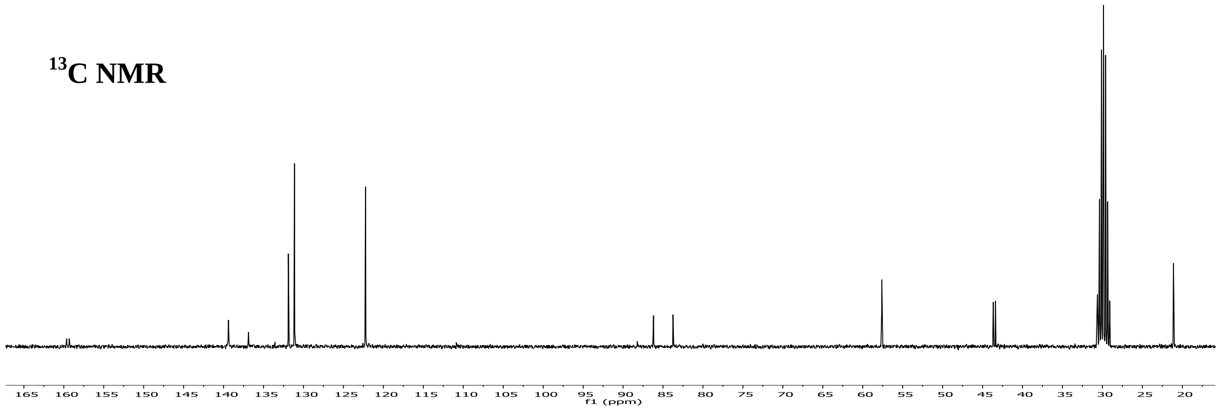

^{19}F NMR

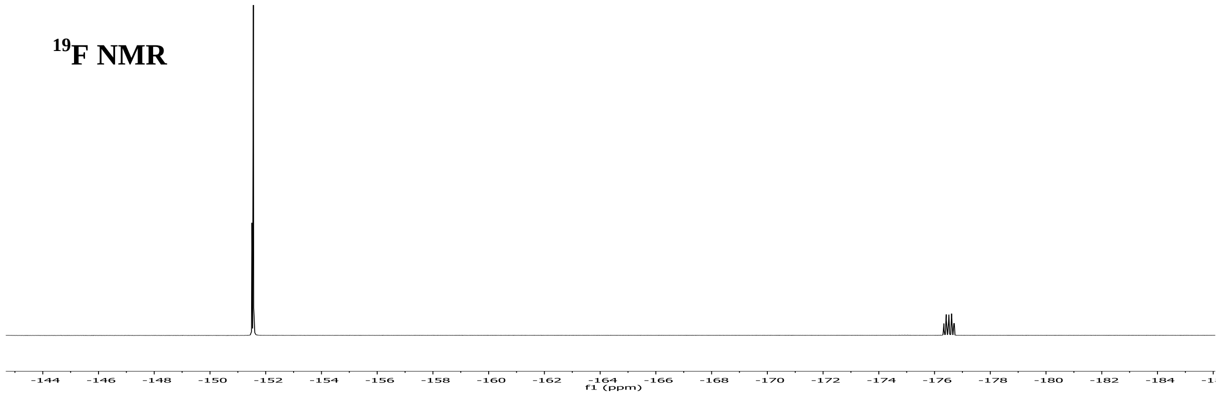

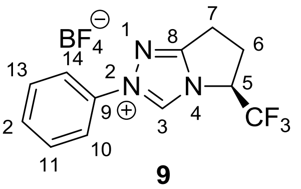

9

^{1}H NMR

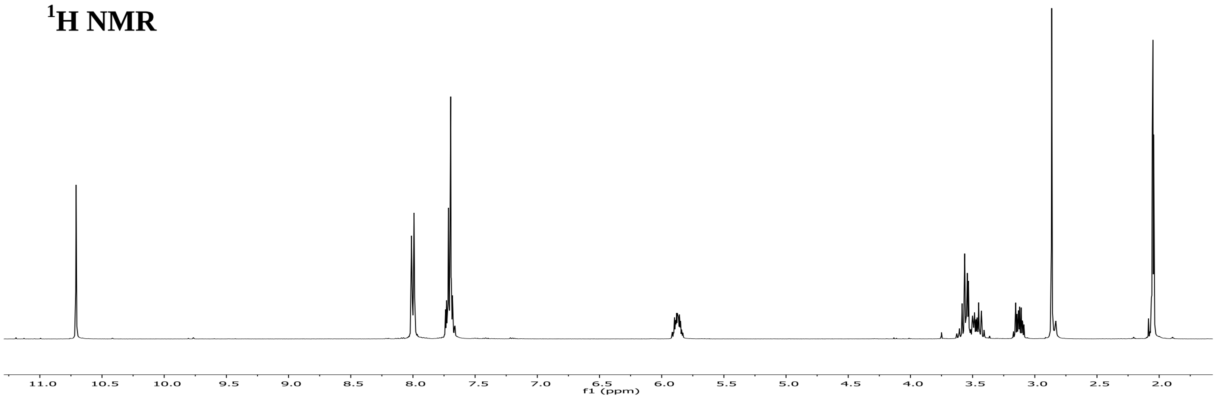

^{13}C NMR

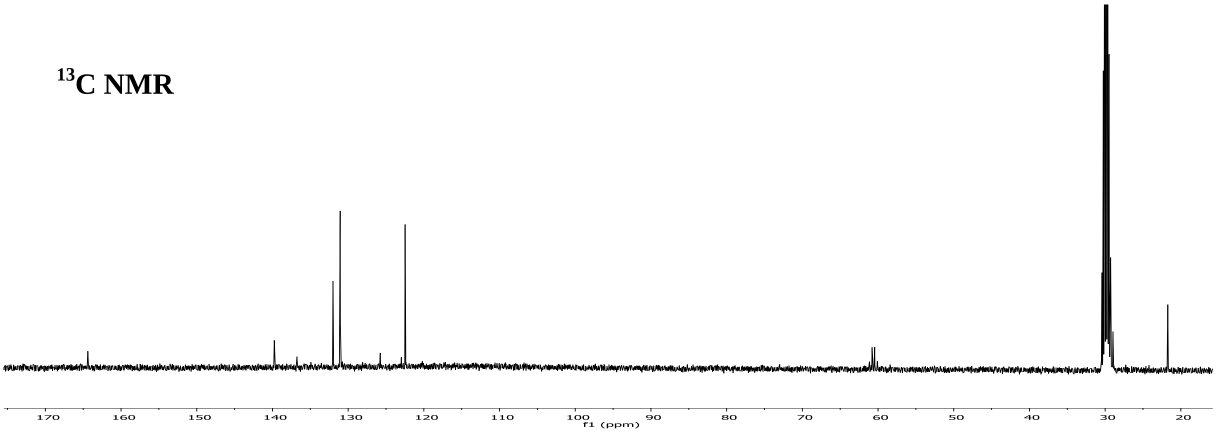

^{19}F NMR

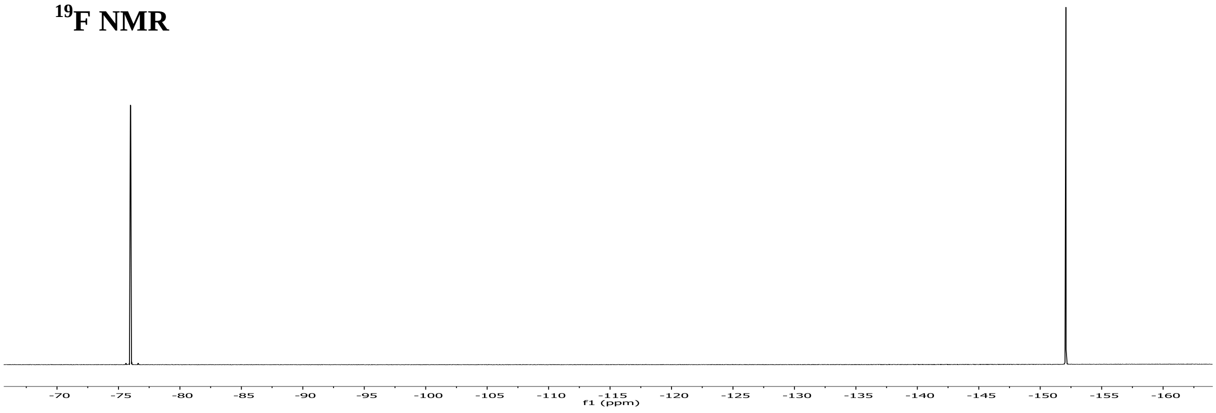

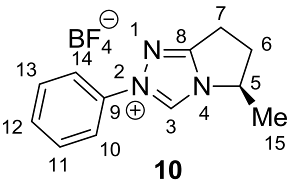

10

^{1}H NMR

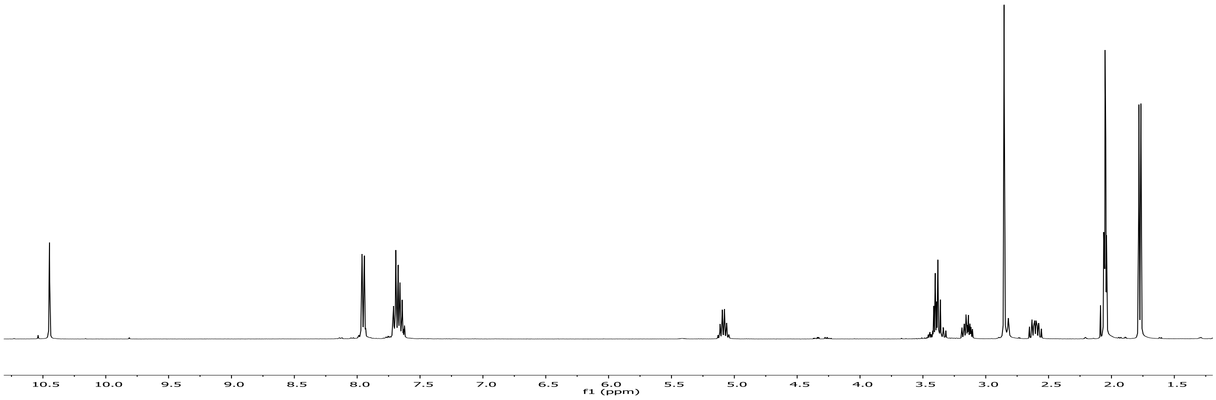

^{13}C NMR

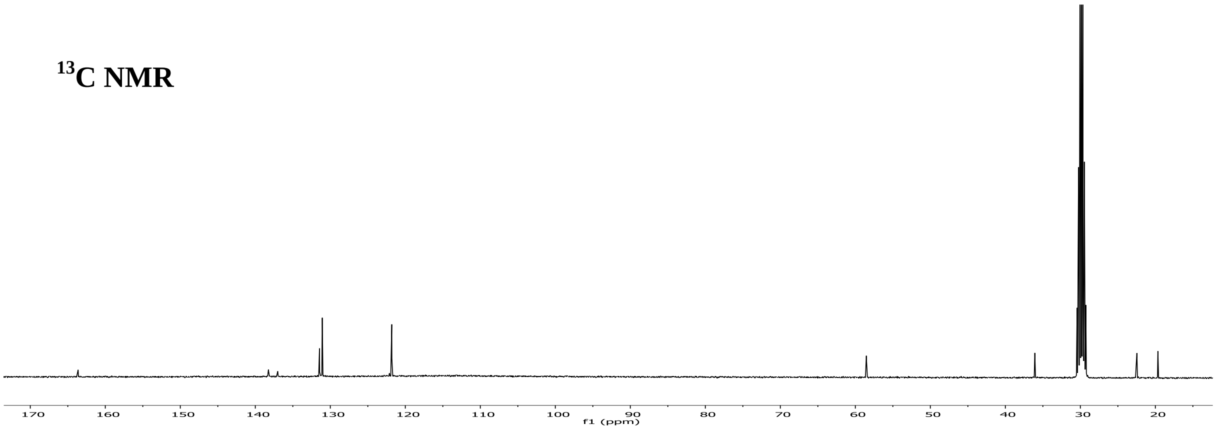

^{19}F NMR

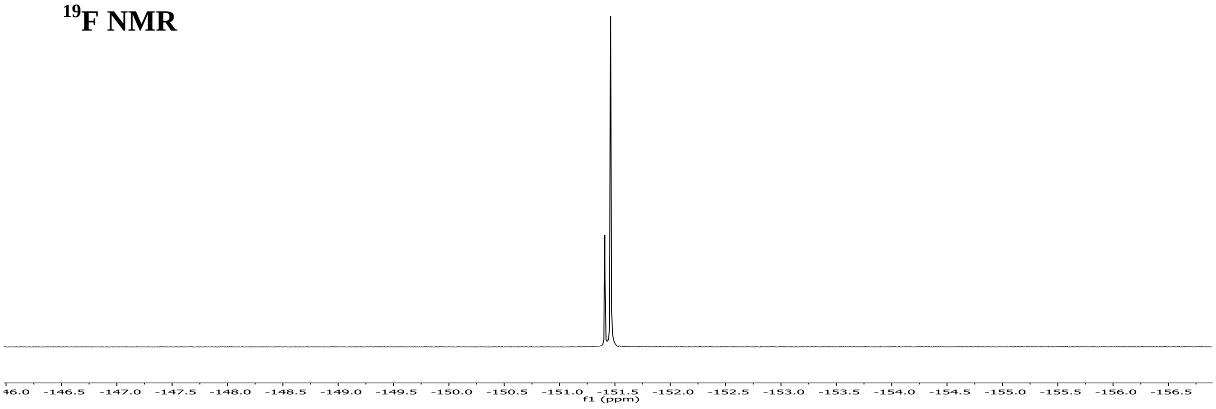

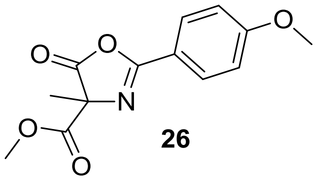

26

^{1}H NMR

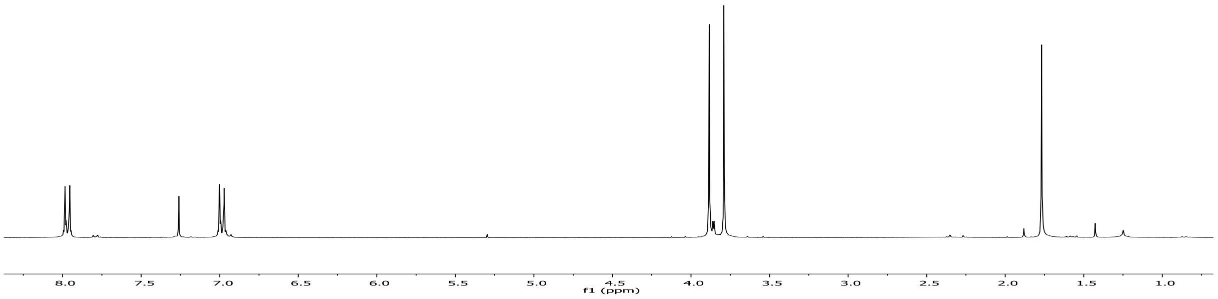

Supplement: File 1 — Experimental part. [file Beilstein_J_Org_Chem-09-2812-s001.pdf]
